# Oncological advantage of nonintubated thoracic surgery: Better compliance of adjuvant treatment after lung lobectomy

**DOI:** 10.1111/1759-7714.13672

**Published:** 2020-09-28

**Authors:** József Furák, Dóra Paróczai, Katalin Burián, Zsolt Szabó, Tamás Zombori

**Affiliations:** ^1^ Department of Surgery University of Szeged Szeged Hungary; ^2^ Department of Pulmonology University of Szeged Deszk Hungary; ^3^ Department of Medical Microbiology and Immunobiology University of Szeged Szeged Hungary; ^4^ Department of Anaesthesiology and Intensive Therapy University of Szeged Szeged Hungary; ^5^ Department of Pathology University of Szeged Szeged Hungary

**Keywords:** Adjuvant chemotherapy, lobectomy, nonintubated, non‐small cell lung cancer, video‐assisted thoracic surgery

## Abstract

**Background:**

Video‐assisted thoracoscopic (VATS) surgery contributes to improved survival, adjuvant chemotherapy delivery and less postoperative complications. Nonintubated thoracic surgery (NITS) VATS procedures improves immunological responses in lung cancer patients; however, there is no data regarding adjuvant chemotherapy delivery effectiveness following NITS lobectomies. In this study, we aimed to compare protocol compliance and toxic complications during adjuvant chemotherapy after intubated and nonintubated VATS lobectomies in non‐small cell lung cancer (NSCLC).

**Methods:**

We retrospectively reviewed the medical records of 66, stage IB–IIIB NSCLC patients who underwent intubated or nonintubated VATS lobectomy and received adjuvant chemotherapy.

**Results:**

A total of 38 patients (17 males, mean age 64 years) underwent conventional VATS and 28 (7 males; mean age 63 years) uniportal VATS NITS. Both groups had comparable demographic data, preoperative pulmonary function, and Eastern Cooperative Oncology Group (ECOG) status. Among the intubated and nonintubated patients, 82% and 75% were diagnosed with adenocarcinoma, respectively. The incidence of adenocarcinoma and squamous cell carcinoma cases were similar in both groups; however, the pathological staging showed significant differences, as 5 (18%) nonintubated patients had stage IB lung cancer, compared with the intubated group (*P* = 0.01). Further distribution of stages was similar between the groups. We observed significant differences in chest tube duration and operation time in the nonintubated group (*P* < 0.01). Among nonintubated patients, 92% completed the planned chemotherapy protocol, compared to 71% of the intubated group (*P* = 0.035). Grade 1/2 toxicity occurred significantly more often in the intubated group (16% vs. 0%, *P* = 0.03) and there was a lower incidence of grade 4 neutropenia in the nonintubated group (0% vs. 16%, *P* = 0.03).

**Conclusions:**

Our results showed that the nonintubated procedure resulted in improved adjuvant chemotherapy compliance and lower toxicity rates after lobectomy.

**Key points:**

**Significant findings of the study:**

- Oncological advantage of the non‐intubated thoracic surgery: better compliance with therapy protocol.

**What this study adds**

- NITS lobectomies contribute to better administration of adjuvant chemotherapy with the planned cycle number and dosage.

## Graphical abstract

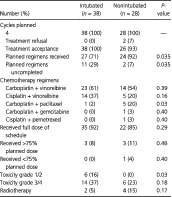

## Introduction

Excessive surgical procedures can cause an altered, uncompensated proinflammatory response to surgical trauma and ventilation; therefore, video‐assisted thoracoscopic surgery (VATS) is regarded as more reliable with a lower number of postoperative complications and morbidity.^1^, ^2^ Unlike thoracotomy, performing VATS can infer several immunological benefits associated with a favorable immune response to surgery. Thoracotomy can result in increased secretion of interleukins and decreased cellular functions that influence postoperative complications, antitumor immunity and infections, similar to other surgical procedures.^3^ Patients undergoing thoracotomy have been reported to show a profound interleukin (IL)‐6, IL‐8, IL‐10 and tumor necrosis factor (TNF)‐α production in serum compared to that in patients undergoing VATS lung resection, which has been shown to affect further proinflammatory properties and host defense against infections.^4^, ^5^ In addition, IL‐6 and IL‐10 plasma concentrations have been shown to be elevated at the time of wound closure and on the first postoperative day, predicting a significant probability of postoperative complications and emerging as a risk factor in these patients.^6^ Furthermore, there are emerging evidences to suggest that there is a relationship between lymphocyte, natural killer (NK) cell count, function, signaling and surgical approach. Thoracotomy has previously been reported to cause a significant reduction in CD4^+^ T cell count and a prolonged NK cell suppression when compared with VATS resection.^7^

Applying a VATS approach seems to be superior to thoracotomy in treating immunocompromised lung cancer patients in order to minimize the risk of postoperative mortality, complications and support the efficacy of adjuvant chemotherapy.^8^ There is increasing evidence that VATS lung resection contributes to better adjuvant chemotherapy compliance, improved tolerance and mitigation of hematological toxicity compared to open thoracic surgery.^9^ Of note, thoracoscopic procedures have also been reported to facilitate the delivery of adjuvant chemotherapy and significant differences in patients have been observed who have received reduced chemotherapy doses or an incomplete protocol compared to thoracotomy.^10^

All these results indicate that there is a demand to develop a less invasive version of VATS surgery to achieve even better surgical outcomes in lung cancer patients: the innovation of uniportal, especially nonintubated VATS surgery, has already been confirmed as being advantageous in early and advanced cases of non‐small cell lung cancer (NSCLC).^11^ Nonintubated uniportal operations have been reported to cause a significantly lower negative impact on NK cells and lymphocytes, resulting in shortened hospital stay and lower morbidity rates.^12^

However, no data is available on whether nonintubated VATS lobectomies are able to achieve better oncological outcomes in adjuvant chemotherapy doses, completed cycles or hematological toxicity. Based on the immunological benefits, in this retrospective study we hypothesized that patients who underwent nonintubated thoracoscopic surgery (NITS) present better protocol compliance to adjuvant chemotherapy in terms of administered dosage and cycle completion. Our aim was to analyze the differences in chemotherapy features, hematological toxicities and postoperative complications between intubated and nonintubated patients after VATS lobectomy.

## Methods

### Study design and patient selection

A retrospective analysis was conducted of 66 IB–IIIB NSCLC patients who received adjuvant chemotherapy (with or without radiotherapy) after undergoing either intubated and nonintubated VATS procedures between January 2014 and December 2019 at the Department of Surgery, University of Szeged. Due to the adjuvant chemotherapy protocols used, patients were treated at the Department of Pulmonology, University of Szeged, Hospital of Chest Diseases, Deszk. Between January 2014 and December 2016, 38 patients underwent standard multiportal VATS lobectomies with general anesthesia under one‐lung mechanical ventilation as previously described.^13^ Between January 2017 and December 2019, nonintubated uniportal VATS lobectomy was performed in 28 patients, as suggested in previous studies.^14^, ^15^ All patients were staged according to the International Association for the Study of Lung Cancer (IASLC) eighth TNM staging guideline for lung cancer^16^ and underwent preoperative chest computed tomography (CT), brain CT/magnetic resonance imaging (MRI), bone scan, abdominal ultrasound/CT, bronchoscopy, pulmonary function testing and laboratory examinations. Positron emission tomography (PET) was not available for all patients. Patients provided a written informed consent to surgery and chemotherapy and the research was approved by the ethics committee of the Hungarian Medical Research Council.

Data were collected from institutional databases and included demographic features, body mass index (BMI), Eastern Cooperative Oncology Group (ECOG) status, smoking habits, pulmonary function testing, histological type, pathological grade, operative time, chest tube duration, postoperative complications and adjuvant chemotherapy characteristics.

### Surgery

#### Patient selection

Oncologically the patient selection was similar in both groups. According to a consensus meeting recommendations for VATS lobectomy, patients with early stage lung cancer (<7 cm, N0, and N1 patients) were scheduled.^17^ The surgical procedure of VATS lobectomy in the intubated and in the nonintubated groups were the same, but for the nonintubated method there were some exclusion criteria because of patient safety: suspicion of difficult intubation, full anticoagulation, reflux disease, cardiac instability or mental problem.^18^ In our current practice, patients with body mass index (BMI) of <30 were indicated for NITS.

#### Surgical procedure

We performed multiportal and uniportal VATS methods for intubated and NITS procedures as previously mentioned in the literature.^19^, ^20^, ^21^ The multiportal procedure was used until June 2015.

For NITS, at the incision site in the fifth intercostal space in the mid‐axillary line, 2% lidocaine (5 mg/kg) was injected subcutaneously, and an incision made. After entering the chest cavity, the lung gradually became atelectatic. An intercostal nerve blockade was performed using 0.5% bupivacaine between the second and fifth intercostal nerves and bupivacaine was also used for the vagus nerve blockade (right side in the upper mediastinum; left side in the aortopulmonary window). After this point, endoscopic instrument manipulation was the same in both groups, but in the nonintubated group the patients were able to breathe spontaneously without mechanical ventilation.

### Anesthesiology

With regard to the NITS surgery, the exclusion criteria are detailed above. In addition to standard monitoring (ECG, O_2_ saturation, noninvasive blood pressure), depth of anesthesia monitoring using the Bispectral Index (BIS; Medtronic Vista) and invasive blood pressure (IBP) measurements were performed. Midazolam and fentanyl were administered prior to surgery. Anesthesia was induced and maintained with closed‐loop titration of propofol administered via target control infusion to maintain the BIS at 40–60 according to the published recommendations.^22^ After an adequate depth of anesthesia was achieved, a laryngeal mask was inserted for airway maintenance. Spontaneous breathing was also maintained throughout and ventilation was monitored using capnography. Oxygen and air mixture were supplemented via a T‐piece and FiO_2_ was titrated to keep the SpO_2_ above 92%.

For intubated cases we performed the gold standard technique, with relaxation and intubation, and one lung ventilation.

#### Adjuvant chemotherapy

Postoperatively, a multidisciplinary team including a pathologist, respiratory physicians, oncologists and thoracic surgeons discussed every histopathology result and decided on the necessity of adjuvant chemotherapy based on former recommendations.^23^, ^24^, ^25^ Patients were staged using the eighth edition TNM staging system and those with ECOG 0 or 1 were discussed and chemotherapy regimens were determined based on the physician's preference. All patients were scheduled to receive four‐cycle platinum‐based combined chemotherapy regimens every three weeks. The majority of agents administered included cisplatin (80 mg/m^2^), or carboplatin (area under curve [AUC] 5) plus vinorelbine (30 mg/m^2^), carboplatin (300 mg/m^2^) plus gemcitabine (1200 mg/m^2^) or paclitaxel (175 mg/m^2^) and cisplatin (70 mg/m^2^) plus gemcitabine (1250 mg/m^2^). Dose reduction, treatment delay or completion were decided by the treating physician using objective criteria (white blood cell count, anemia, serum creatinine, gastrointestinal or neurological symptoms) and performance status as subjective criteria. Sequential radiotherapy was administered in selected patients. Laboratory testing was implemented before each cycle to detect hematological, renal or hepatic failure. At least 1.5 g/L absolute neutrophil count was required to continue the therapy, and in the case of patients with grade 3 or 4 neutropenia, granulocyte colony stimulating factor (G‐CSF) was used according to the American Society of Clinical Oncology (ASCO) guidelines. Follow‐up visits were performed in the outpatient ward at the Department of Pulmonology, University of Szeged, Hospital of Chest Diseases every three, six, and 12 months after the last dose of chemotherapy was administered.

#### Statistical analysis

Patient characteristics and chemotherapy protocol compliance, toxicity, postoperative complications, and survival data were compared between the intubated and nonintubated groups. As data were not normally distributed, we used Student's *t*‐test and Wilcoxon Mann‐Whitney two‐sample test. All statistical analyses were performed using SigmaPlot for Windows Version 12.0 software. A value of *P* < 0.05 indicated a statistically significant difference.

## Results

### Patient characteristics

There was no significant differences in age, gender, BMI, ECOG status and smoking habits in all patients who underwent either intubated or nonintubated VATS lobectomies. There were also no significant differences in hematological and pulmonary diseases, arrhythmia, former malignancies, or chronic kidney failure which have a negative impact on further oncological outcomes. We used the modified, age‐adjusted Charlson Comorbidity Index (ACCI) to evaluate differences between the two groups with regard to comorbidities and weighted based on patients' age, as it has previously been described that ACCI is a better predictor factor of survival in lung cancer patients who have undergone surgery. Using ACCI, we did not detect any significant differences.^26^ Baseline pulmonary function testing showed no significant differences in FEV1 and DLCO between intubated and nonintubated individuals, in accordance with the lack of differences in the incidence of COPD or asthma. Further baseline clinical characteristics are provided in Table 1.

**Table 1:** Main clinical characteristics and demographic data of patients who underwent intubated and nonintubated VATS lobectomies

	Intubated (*n* = 38)	Nonintubated (*n* = 28)	*P*‐value
Mean age (median)	64 (63)	63.03 (63)	0.075
Gender (n, %)	Male:17 (45),	Male: 7 (25)	—
	Female: 21 (55)	Female: 21 (75)	
BMI (kg/m^2^)	24.83 ± 3.07	24.31 ± 4.17	0.28
ECOG (n, %)			0.40
0	28 (74)	1 (68)	
1	10 (26)	9 (32)	
Smoking status (No.%)	Never: 12 (32)	Never: 10 (36)	0.46
	Previously: 14(36)	Previously: 5 (18)	0.07
	Mean py = 33	Mean py = 30.6	
	Habitual: 12(32)	Habitual: 13 (46)	0.16
	Mean py = 30	Mean py = 35	
Preoperative complications No. (%)			
CHD	10 (26)	4 (14)	0.04
Hypertension	23 (60)	9 (32)	0.02
COPD or asthma	9 (24)	10 (36)	0.21
Former malignancy	3 (8)	4 (14)	0.33
Hematological diseases	3 (8)	2 (7)	0.64
Chronic kidney failure	3 (8)	1 (3.5)	0.43
Arrhythmia	6 (16)	4 (14)	0.57
Diabetes mellitus	4 (10)	2 (7)	0.49
ACCI (mean ± SD)	5.56 ± 1.4	5.18 ± 1.62	0.20
FEV1 (L, mean ± SD)	2.26 ± 0.83	2.16 ± 0.69	0.33
FEV1 (%, mean ± SD)	87.45 ± 20.24	87.54 ± 19.24	0.49
DLCO (%, mean ± SD)	64.15 ± 18.45	68.92 ± 16.43	0.20
Pathological types No. (%)			
Adenocarcinoma	31 (82)	21 (75)	0.36
Squamous cell carcinoma	5 (13)	5 (18)	0.42
Other	2 (5)	2 (7)	0.56
Pathological staging No. (%)			
IB	0 (0)	5 (18)	0.01
IIA	10 (26)	5 (18)	0.30
IIB	13 (34)	7 (25)	0.29
IIIA	15 (40)	10 (36)	0.47
IIIB	0 (0)	1 (3)	0.42
IIIC	0 (0)	0 (0)	—
IVA	0 (0)	0 (0)	—
IVB	0 (0)	0 (0)	—

ACCI, age‐adjusted Charlson Comorbidity Index; BMI, body mass index; CHD, chronic heart disease; COPD, chronic obstructive pulmonary disease; py, pack‐year; SD, standard deviation.

The incidence of adenocarcinoma and squamous cell carcinoma cases were similar in both groups; however, the pathological staging showed significant differences, as 18% of nonintubated patients had IB lung cancer, compared to the intubated group (*P* = 0.01) (Table 1). We observed no differences in the type of anatomic pulmonary resections (Table 2).

**Table 2:** Anatomic pulmonary resections

Number (%)	Intubated (*n* = 38)	Nonintubated (*n* = 28)	*P*‐value
Right upper lobectomy (RUL)	12 (32)	13 (46)	0.30
Right middle lobectomy (RML)	4 (10.5)	2 (7)	0.47
Right lower lobectomy (RLL)	4 (10.5)	3 (11)	0.65
Left upper lobectomy (LUL)	10 (26)	6 (22)	0.40
Left lower lobectomy (LLL)	8 (21)	4 (14)	0.33

LLL, left lower lobectomy; LUL, left upper lobectomy; RLL, right lower lobectomy; RML, right middle lobectomy; RUL, right upper lobectomy.

### Adjuvant chemotherapy protocol compliance and toxicity

All patients who were considered for the four‐cycle adjuvant chemotherapy regimen underwent lobectomy; however, treatment refusal occurred in two cases among the nonintubated patients. A total of 38 patients in the intubated group and 26 patients in the nonintubated group received platinum‐based chemotherapy combined with mostly vinca‐alkaloid, taxane or gemcitabine. Significantly, more carboplatin and taxane combinations was administered in the nonintubated group (*P* = 0.03), than other chemotherapy regimens. Among the nonintubated and intubated patients, 85% and 92%, respectively, received full dose schedule, meaning that patients were treated with a full dose of chemotherapy regimens based on their body surface or AUC 5 value in each cycle. The full dose schedule is the received dose in mg in each cycle, calculated based on patients' demographic and laboratory data (weight, height and renal function parameters). Dosage in mg of adjuvant chemotherapy was initially determined and not altered significantly by the multidisciplinary team and oncologists, unlike the number of cycles. Dose reductions occurred in patients with grade 3 or 4 toxicities and were performed in each group without significant differences. Radiotherapy was implemented sequentially in 5% of the intubated and 15% of the nonintubated group without significant differences (Table 3).

**Table 3:** Main general states of patients who received adjuvant chemotherapy

Number (%)	Intubated (*n* = 38)	Nonintubated (*n* = 28)	*P*‐value
Cycles planned			
4	38 (100)	28 (100)	—
Treatment refusal	0 (0)	2 (7)	
Treatment acceptance	38 (100)	26 (93)	
Planned regimens received	27 (71)	24 (92)	0.035
Planned regimens uncompleted	11 (29)	2 (7)	0.035
Chemotherapy regimens			
Carboplatin + vinorelbine	23 (61)	14 (54)	0.39
Cisplatin + vinorelbine	14 (37)	5 (20)	0.16
Carboplatin + paclitaxel	1 (2)	5 (20)	0.03
Carboplatin + gemcitabine	0 (0)	1 (3)	0.40
Cisplatin + pemetrexed	0 (0)	1 (3)	0.40
Received full dose of schedule	35 (92)	22 (85)	0.29
Received >75% planned dose	3 (8)	3 (11)	0.46
Received <75% planned dose	0 (0)	1 (4)	0.40
Toxicity grade 1/2	6 (16)	0 (0)	0.03
Toxicity grade 3/4	14 (37)	6 (23)	0.18
Radiotherapy	2 (5)	4 (15)	0.17

Nevertheless, significantly more grade 1 or 2 toxic symptoms including nausea, vomiting, anemia, thrombocytopenia, and neutropenia were reported in the intubated group (*P* = 0.03). Additionally, grade 4 neutropenia requiring G‐CSF treatment was more likely to occur in the participants in the intubated group, compared to those in nonintubated group, showing no incidence (*P* = 0.03). Grade 3 or 4 anemia and thrombocytopenia were not experienced in a statistically significant proportion between the groups (Table 4).

**Table 4:** Grade 3/4 hematological toxicity in patients who received adjuvant chemotherapy

Number (%)	Intubated	Nonintubated	*P*‐value
Grade 3 neutropenia	6 (16)	5 (19)	0.48
Grade 4 neutropenia	6 (16)	0 (0)	0.03
Grade 3 anemia	1 (3)	1 (4)	0.65
Grade 4 anemia	0 (0)	0 (0)	—
Grade 3 thrombocytopenia	1 (3)	0 (0)	0.59
Grade 4 thrombocytopenia	0 (0)	0 (0)	—

A total of 51 (80%) patients completed the planned four‐cycle platinum‐based chemotherapy according to protocols. Only 71% of patients in the intubated group reached complete cycles, compared to 92% of the nonintubated group, showing statistically significant differences (*P* = 0.035). Therapy was discontinued in case of severe adverse effects or patients refused further treatment (Table 5).

**Table 5:** Protocol compliance in case of treatment acceptance

Number (%)	Intubated	Nonintubated	*P*‐value
Cycle I	38 (100)	26 (100)	—
Cycle II	35 (92)	26 (100)	0.2
Cycle III	34 (89)	24 (92)	0.53
Cycle IV	27 (71)	24 (92)	0.035

### Surgical and survival data analysis

Significantly less operation time was required for performing the nonintubated surgical technique than the intubated technique (91.04 ± 23.88 vs. 125.77 ± 38.07 minutes, *P* < 0.01). Chest tube duration analysis showed that patients without intubation required the tube for significantly fewer days, compared to those in the intubated group (2.12 ± 1.16 vs. 4.33 ± 3.58, *P* < 0.01). Moreover, postoperative complications, mainly subcutaneous emphysema, were encountered with higher incidence in patients in the intubated group (*P* = 0.027) (Table 6).

**Table 6:** Postoperative results

	Intubated	Nonintubated	*P*‐value
Operation time (minutes)	125.77 ± 38.07	91.04 ± 23.88	<0.01
Days of chest tube	4.33 ± 3.58	2.12 ± 1.16	<0.01
Postoperative complications			
Subcutaneous emphysema	4 (10.5)	0 (0)	0.027
Fever	0 (0)	0 (0)	
Reoperation	2 (5.2)	0 (0)	

We detected no postoperative mortality in either group. Patients who received 1–4 cycles of chemotherapy showed no significant differences in actual survival during follow‐up (Table 7).

**Table 7:** Survival data (*n* = 66)

Number (%)	Intubated	Nonintubated	*P*‐value
Postoperative mortality	0 (0)	0 (0)	—
Actual survival			
Alive	30 (79)	26 (93)	0.11
Deceased during follow‐up	8 (21)	2 (7)	

## Discussion

In this retrospective study, we compared oncological and surgical features of 66 patients who had undergone either intubated or nonintubated VATS lobectomies. There is increasing evidence that minimally invasive techniques result in better outcomes with regard to hospital stay, blood loss, preserved pulmonary functions, immune responses and there may even be oncological advantages.^27^, ^28^, ^29^ VATS surgery contributes to the delivery of adjuvant chemotherapy and a better compliance with therapy protocols. Teh *et al*. found an improved compliance, with earlier initiation of adjuvant platinum plus vinorelbine chemotherapy and a reduction in hematological impact in NSCLC patients after VATS resections compared to thoracotomy.^9^ Similarly, patients receiving docetaxel‐carboplatin adjuvant chemotherapy after VATS surgery gained a well‐tolerated protocol compliance with more completion of the planned cycles.^30^ Moreover, thoracoscopic pulmonary resections and lobectomies have been reported to be associated with significantly fewer dose reductions in patients and delays in therapy schedules.^10^, ^31^ Cisplatin plus gemcitabine, cisplatin plus vinorelbine and carboplatin plus paclitaxel combined chemotherapy regimens also showed better influence on therapy dosage, completed cycles and toxicity in case of NSCLC patients who underwent VATS.^32^ In accordance with previous studies, we found that patients who were treated with nonintubated approach had further remarkable benefits when administered adjuvant chemotherapy. Compared to the conventional VATS technique, after uniportal nonintubated lobectomies we detected a higher proportion of patients who received full four‐cycle regimens, resulting in better protocol compliance.

The avoidance of general anesthesia and one‐lung mechanical ventilation could result in a better physiological inflammatory response, a better conserved lymphocyte and NK cell population; furthermore, the extent of surgical invasiveness has been shown to play a key role in immune response to surgery.^4^, ^33^, ^34^, ^35^ It is well established that a thoracoscopic approach has immunological and clinical advantages over thoracotomy; however, few data exist on the long‐term oncological effects in patients in which a nonintubated VATS procedure has been performed. As the secretion of interleukins, especially IL‐10, are able to mediate an escape of tumor cells from the host immune system and inhibit NK‐cell mediated cytotoxicity, it could partly explain tumor recurrence and the poor prognosis associated with thoracotomy.^36^, ^37^ Therefore, a nonintubated procedure should bring about cellular immune response benefits, a reduction in morbidity, shorter hospital stay and reduced postoperative thorax drainage compared to the conventional VATS technique.^12^, ^38^ A recent study proved that nonintubated lung metastasectomy resulted in fewer immune‐depressive effects, with a lower morbidity rate, and better survival after NITS in malignant pleural effusion cases.^39^

Consequently, we hypothesized that as NITS may have less impact on cellular responses in patients that it might provide further oncological and clinical advantages. We evaluated the dosage on schedule, compilation of planned chemotherapy cycles and toxicity to reach conclusions in terms of protocol compliance. We detected significant toxicity differences between the patients in the intubated and nonintubated groups, since a greater percentage of patients who underwent conventional VATS showed grade 1/2 toxicity and grade 4 neutropenia compared with those who underwent NITS. Chest tube duration and operation time were also significantly reduced in patients who underwent NITS, and that could have an enormous impact on hospitalization and postoperative complications. Theoretically, these differences could be attributable to immune factors influenced by minimally invasive techniques. If so, further randomized studies are needed to reach a conclusion regarding the association between the lack of intubation procedure and long‐term oncological outcomes.

To our knowledge, this is the first study that has revealed a better response to chemotherapy after nonintubated video‐assisted lobectomies over traditional intubated VATS lobectomies. However, there are some limitations in our study because of the limited number of patients included and its retrospective nature. Our results are consistent with former findings and support the efficacy of minimal invasiveness on adjuvant chemotherapy; nevertheless, this is still a controversial field due to the limited clinical data available. Based on our observations, we highlight the necessity of further clinical and basic studies to reach proper conclusions regarding associations between inflammatory, cellular responses and clinical benefits of adjuvant chemotherapy following nonintubated video‐assisted lobectomy.

## Disclosure

The authors declare that they have no conflicts of interest.
